# The Environmental Impacts of Electronic Medical Records Versus Paper Records at a Large Eye Hospital in India: Life Cycle Assessment Study

**DOI:** 10.2196/42140

**Published:** 2024-02-06

**Authors:** Cordelia Kwon, Lernik Essayei, Michael Spencer, Tom Etheridge, Rengaraj Venkatesh, Natrajan Vengadesan, Cassandra L Thiel

**Affiliations:** 1 Department of Population Health NYU Langone Health New York, NY United States; 2 NYU Wagner School of Public Service New York, NY United States; 3 Rausser College of Natural Resources University of California, Berkeley Berkeley, CA United States; 4 EarthShift Global Corvallis, OR United States; 5 Aravind Eye Care System Pondicherry India; 6 Center for Healthcare Innovation and Delivery Science Department of Population Health NYU Langone Health New York, NY United States; 7 Department of Ophthalmology NYU Langone Health New York, NY United States

**Keywords:** carbon emissions, electronic health records, electronic medical records, environmental impact, greenhouse gases, life cycle assessment, low middle income country, medical records, paper medical records, sustainability

## Abstract

**Background:**

Health care providers worldwide are rapidly adopting electronic medical record (EMR) systems, replacing paper record-keeping systems. Despite numerous benefits to EMRs, the environmental emissions associated with medical record-keeping are unknown. Given the need for urgent climate action, understanding the carbon footprint of EMRs will assist in decarbonizing their adoption and use.

**Objective:**

We aimed to estimate and compare the environmental emissions associated with paper medical record-keeping and its replacement EMR system at a high-volume eye care facility in southern India.

**Methods:**

We conducted the life cycle assessment methodology per the ISO (International Organization for Standardization) 14040 standard, with primary data supplied by the eye care facility. Data on the paper record-keeping system include the production, use, and disposal of paper and writing utensils in 2016. The EMR system was adopted at this location in 2018. Data on the EMR system include the allocated production and disposal of capital equipment (such as computers and routers); the production, use, and disposal of consumable goods like paper and writing utensils; and the electricity required to run the EMR system. We excluded built infrastructure and cooling loads (eg. buildings and ventilation) from both systems. We used sensitivity analyses to model the effects of practice variation and data uncertainty and Monte Carlo assessments to statistically compare the 2 systems, with and without renewable electricity sources.

**Results:**

This location’s EMR system was found to emit substantially more greenhouse gases (GHGs) than their paper medical record system (195,000 kg carbon dioxide equivalents [CO_2_e] per year or 0.361 kg CO_2_e per patient visit compared with 20,800 kg CO_2_e per year or 0.037 kg CO_2_e per patient). However, sensitivity analyses show that the effect of electricity sources is a major factor in determining which record-keeping system emits fewer GHGs. If the study hospital sourced all electricity from renewable sources such as solar or wind power rather than the Indian electric grid, their EMR emissions would drop to 24,900 kg CO_2_e (0.046 kg CO_2_e per patient), a level comparable to the paper record-keeping system. Energy-efficient EMR equipment (such as computers and monitors) is the next largest factor impacting emissions, followed by equipment life spans. Multimedia Appendix 1 includes other emissions impact categories.

**Conclusions:**

The climate-changing emissions associated with an EMR system are heavily dependent on the sources of electricity. With a decarbonized electricity source, the EMR system’s GHG emissions are on par with paper medical record-keeping, and decarbonized grids would likely have a much broader benefit to society. Though we found that the EMR system produced more emissions than a paper record-keeping system, this study does not account for potential expanded environmental gains from EMRs, including expanding access to care while reducing patient travel and operational efficiencies that can reduce unnecessary or redundant care.

## Introduction

### Expansion of Electronic Medical Records

Health care systems are in the middle of a rapidly shifting framework, one that comes not from new medicines or new gene therapies but from the way medical information is tracked, stored, and shared through electronic health records (or electronic medical records [EMRs]). The use of EMR has been steadily increasing across the world since the early 2000s [1,2].

The COVID-19 pandemic and the subsequent rise of telemedicine propelled the expansion of EMRs. Telemedicine visits have expanded across various medical settings and are becoming more common in low- and middle-income countries (LMICs) [3]. In order to better integrate video visits and allow important information to be shared between doctor and patient remotely, patients and physicians began increasingly relying on EMRs to integrate video visits and allow important information to be virtually shared between doctor and patient, as well as among other providers in the patient’s circle of care, ensuring better informed care and better outcomes [4]. EMRs also facilitated the implementation of screening processes for COVID-19, reporting and analytics of COVID-19 cases, and other outbreak management supports [5].

Beyond facilitating telemedicine, there is a sizable body of literature on the impacts, mostly beneficial, of EMR transitions on physician use, patient experience, and hospital efficiency [6-10]. EMRs are key data management tools as well as important validation tools for reducing clinician error [11]. When used in conjunction with telemedicine, they can facilitate medical care in rural and remote areas, which is particularly important in LMICs [3]. On the other hand, EMR adoption and implementation require immense institutional support, such as policy making and cultural change.

However, these considerations only represent some of the implications of implementing EMR systems. The carbon footprint of health care is 4.4% of net emissions globally [12], and therefore considering the environmental impact of implementing EMR systems is key to understanding the implications of their use. It is also worth noting that >90% of US hospitals have adopted some kind of certified EMR system to conduct 7 out of 9 patient care-related processes [13]. Although the rate of adoption of EMR systems in other countries, particularly LMICs, is slower due to the high costs associated with their purchase and maintenance, technological advancement and easier implementation have led to a steady upward trend, and it is expected that other countries’ systems will soon mirror the United States. Understanding these implications is even more important when considering implementing EMR systems in the rapidly growing health care systems of LMICs. Measures to reduce carbon emissions in LMICs’ health systems would not only combat climate change, which disproportionately threatens the health of the most vulnerable populations, but would also garner long-term savings [14].

To date, there is relatively little quantitative data about the environmental implications of transitioning to an EMR system, and even less in the context of LMICs. Transitioning away from paper-based records has the potential to reduce emissions from paper production and waste. However, electronic records also require infrastructure and electricity to implement and maintain, which require carbon emissions. This study will therefore try to fill this gap in knowledge by looking at how the transition to EMRs impacts a private institution in an LMIC, as well as addressing wider relevance toward sustainable and green health care goals.

### Case Location

Aravind Eye Care Systems (referred to as “Aravind” henceforth) is the largest conglomerate eye care provider in India. It annually handles over 4 million outpatient visits and performs over 400,000 surgeries or laser procedures, with over 50% of them being free or steeply subsidized. Aravind’s operating theaters are highly productive through efficient assembly line service operations and effective resource consumption. Cataract surgeries at Aravind are noted for their excellent outcomes, efficiency, cost-effectiveness, and environmental sustainability [15-18]. One sustainability initiative was the system’s transition from paper-based health records, which was enacted at Aravind’s Pondicherry-based location in 2018. Aravind-Pondicherry tertiary center is a 37,160 m^2^ facility located on an 81,000 m^2^ plot. It has 650 beds and caters to the needs of over 21.2 million people in the nearby districts of Tamil Nadu and Pondicherry. Aravind-Pondicherry served 568,982 patients in 2016, while still on a paper medical record system, and 538,325 in 2019, after switching to an EMR system. This study compares the life-cycle greenhouse gas emissions (GHGs) of their paper record-keeping system in 2016 with their EMR system in 2019, 1 year after it was enacted.

## Methods

### Overview of Life Cycle Assessment

This study uses life cycle assessment (LCA) to estimate the GHG emissions of Aravind’s paper record-keeping system (2016) compared with its EMR system (2019). LCA is a tool used to estimate the environmental emissions of a product or process throughout its life cycle, from raw material extraction, production, use, end of life, and all the transportation steps in between. It has been used to estimate emissions for a variety of products and services, including in the health care space [19,20]. Though it has been increasingly used to monitor the impact of telemedicine, this is the first time, to our knowledge, that it has been used to estimate GHGs from EMR systems.

**Figure 1:**
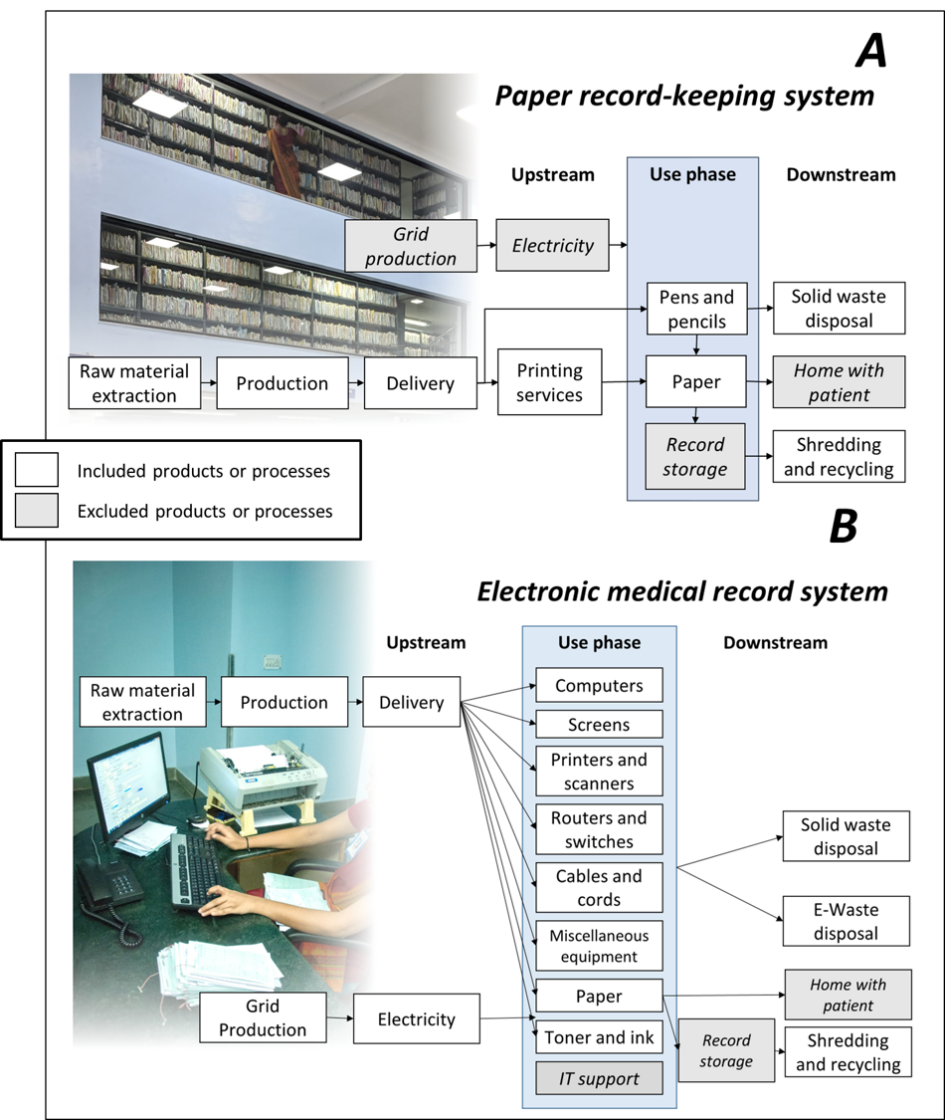
System boundaries and included or excluded elements. (A) Aravind Eye Care System’s paper record-keeping system (before 2016) and (B) Aravind’s electronic medical record (EMR) system (implemented in 2018 and tested in 2019).

According to ISO (International Organization for Standardization) 14040 standard [21], LCA is conducted in four steps: (1) goal and scope definition, (2) life cycle inventory (LCI), (3) life cycle impact assessment (LCIA), and (4) interpretation. The goal of this study is to compare the environmental impacts of Aravind’s paper record-keeping system with their EMR system (Figures 1A and 1B), annually and on a per-patient basis. The functional unit is the creation and maintenance of 1 patient’s health records. We include all production and disposal impacts from disposable and reusable supplies and equipment in both systems. We did not include heating, ventilation, and air conditioning required to maintain either system, given the difficulty in making such an allocation. Space is a major issue for Aravind’s paper record-keeping system, for which this study may not accurately estimate the emissions. Aravind’s policy requires them to store paper records on site for 3 years after the patient’s last visit for outpatient records and 5 years for surgical records. As of 2020, Aravind-Pondicherry was maintaining 1,600,000 patient paper files in 2 hospital spaces, for a total of 237 m^2^ of air-conditioned space dedicated to paper record storage. Likewise, EMRs require cooling servers for data storage, though this would take up considerably less space per patient than paper records.

### Inventory: Consumable Supplies

Aravind’s paper medical records required pens, pencils, and paper forms. The study team obtained purchase records for all paper, pens, pencils, and toner purchased by Aravind in 2016 and in 2019. The excess of pens and pencils purchased in 2016 was assumed to be a requirement of paper record-keeping (ie, the quantity purchased in 2016 was subtracted from the quantity purchased in 2019 to assess the changes due to the EMR implementation). Our LCI databases did not contain unit processes for pens or pencils, so we estimated their GHG emissions from previous literature [22-24]. To estimate the environmental emissions from disposing of these items, we converted the number of items purchased into weights by directly measuring the weight of a generic pen and pencil. We assumed these products were disposed of in a sanitary landfill.

Paper purchasing data were obtained for 2016 and 2019. All forms for the paper medical record were printed by a local third party using offset printing methods. After enacting the EMR, some paper forms were still printed by Aravind in the hospital on purchased laser printers. For example, a patient consent form and medical documents are often sent home with patients. Though Aravind purchased an extra 430 toner cartridges in 2019 compared with 2016, these are not expected to be the annual purchases of toner required for the EMR system. The capacity of the cartridges purchased ranges from 1600 to 3500 pages printed, so we conservatively use the 1600-page estimate. As Aravind used 251,180 sheets of paper for their EMRs, this resulted in an estimated 157 cartridges expended.

For disposal impacts of these consumables, we did not assume any losses in weight as pens or pencils were used, but we did account for the “loss” of toner in the cartridges at their end of life by using empty cartridge specifications from the manufacturer. Aravind shreds all paper records and sells the shreds to local paper dealers for recycling. We assumed all papers were disposed of this way, even if some were taken home by patients.

### Inventory: EMR Capital Outlay and Disposal

Installing the EMR system required the purchase of capital equipment and wiring infrastructure, including computers (tablets and desktops), screens, scanners, printers, routers and switches, cables and chords, other miscellaneous equipment (platforms, racks, power strips, monitor stands), and IT support. Aravind supplied a list of this equipment, its make and model, and its costs. The production and disposal impacts of these items need to be allocated across product life spans. The life span of computers and their electrical components is highly variable and dependent upon disk size, RAM availability, the condition of their hardware and systems, malware, and initial quality, as well as cultural and community factors. Here, we assume a replacement rate or product lifetime of 5 years for computers [25,26], 4 years for printers and scanners [27], 5.7 years for routers and switches [28], 9.5 years for computer screens [29], and 10 years for cables and miscellaneous equipment. Disposal impacts were estimated based on the weight of the electronic product, according to the manufacturer’s product specifications. We assumed all electronic equipment was sent to e-waste recycling and all consumables were sent to a sanitary landfill.

### Inventory: Energy Consumption

To estimate the energy consumed while using the EMR, we directly measured the kWh consumed by desktop computers and screens using a watt meter, and we used the manufacturer’s product specifications to estimate the power consumption of remaining capital goods such as laptops or tablets, printers, scanners, and routers. We assumed cables and miscellaneous equipment consumed no additional wattage. As a reflection of the average work week and power-down policies at Aravind, we assumed that computers and digital screens are on 10 hours per day and turned off 14 hours per day, 7 days a week. This will likely overestimate their energy consumption, as Aravind typically sees fewer patients on Sundays. These assumptions were validated by Aravind’s staff, and variations to these assumptions were tested in our sensitivity analyses. Routers and switches are rarely powered off at the end of a workday, so their assumed usage is 24 hours per day. For the printers, we assume they are in “printing” mode for 15 minutes each day and in “ready or on” the rest of the 24-hour day. As noted above, the energy required to cool this equipment was not included in this study. We assumed all power was drawn from the local electric grid, using the Indian national average mix of power generation sources.

For data storage, Aravind purchases 500 GB annually from the Google Cloud Platform. The energy consumption of cloud-based data storage is highly variable. In addition, Google Cloud Platform claims to be carbon neutral using carbon offsets [30]. For the purposes of this study, we estimated the power consumption of a typical hard disk using manufacturer specifications and multiplied this wattage (8.5 W) over a year. This will likely overestimate the energy consumption and emissions associated with data storage, particularly for medical records, which are not being constantly modified as other cloud-based data may be.

### Life Cycle Inventory and Impact Assessment Methods

All LCI was created using SimaPro (version 9.3.0.2; Pré Consultants) [31] and the Ecoinvent (version 3.8; Ecoinvent) database [32], one of the most comprehensive LCA databases available, using an allocation and cut-off by classification approach. Two exceptions are the production of pens and pencils. A full list of LCI and chosen unit processes can be found in Table S1 in Multimedia Appendix 1 [31]. The LCIA step was conducted using the US Environmental Protection Agency’s Tool for Reduction and Assessment of Chemicals and Other Environmental Impacts (TRACI; 2.1 version 1.06/US 2008; US EPA) [33]. Here, we report units of GHG emissions in kg carbon dioxide equivalents (CO_2_e). Other emission categories, such as acidification, eutrophication, and air pollution, can be found in Multimedia Appendix 1.

### Sensitivity Analyses and Monte Carlo Assessment

To test our assumptions and account for variations in practices outside of Aravind, we conducted multiple best- and worst-case sensitivity analyses. For the EMR, we analyzed the impact of shorter and longer life spans for capital equipment, more or less energy efficient capital equipment, and the use of solar power instead of the Indian electric grid (energy estimates from previous literature and from Ecoinvent [32]). Table S2 in Multimedia Appendix 1 contains a detailed list of sensitivity assumptions. In addition, we constructed a Monte Carlo assessment (MCA) through SimaPro software to estimate the uncertainty in our models. The unit processes we select to represent the LCI stage are representative of industry averages rather than our specific use case. For example, the unit process for computer manufacturing selected from Ecoinvent represents the manufacturing and shipping of computers globally. The manufacturer used by Aravind could be emitting more or less than the industry average. The MCA allows us to account for that uncertainty. Using 1000 runs and a 95% CI, we assessed the differences in emissions categories between Aravind’s paper record-keeping system and their EMR system, with the Indian grid and with solar power. The MCA, however, excludes impacts from the production of pens and pencils (the data for which came from non-Ecoinvent sources) and disposal pathways. The detailed results of the MCA can be found in Multimedia Appendix 1.

### Ethical Considerations

This study was deemed "non-human subjects research" by Aravind Eye Care System and thus did not require ethical review board approval. No human participants were used in this study, and any data reporting the number of patients served by this system were provided, anonymized, and aggregated by the study location.

## Results

**Figure 2:**
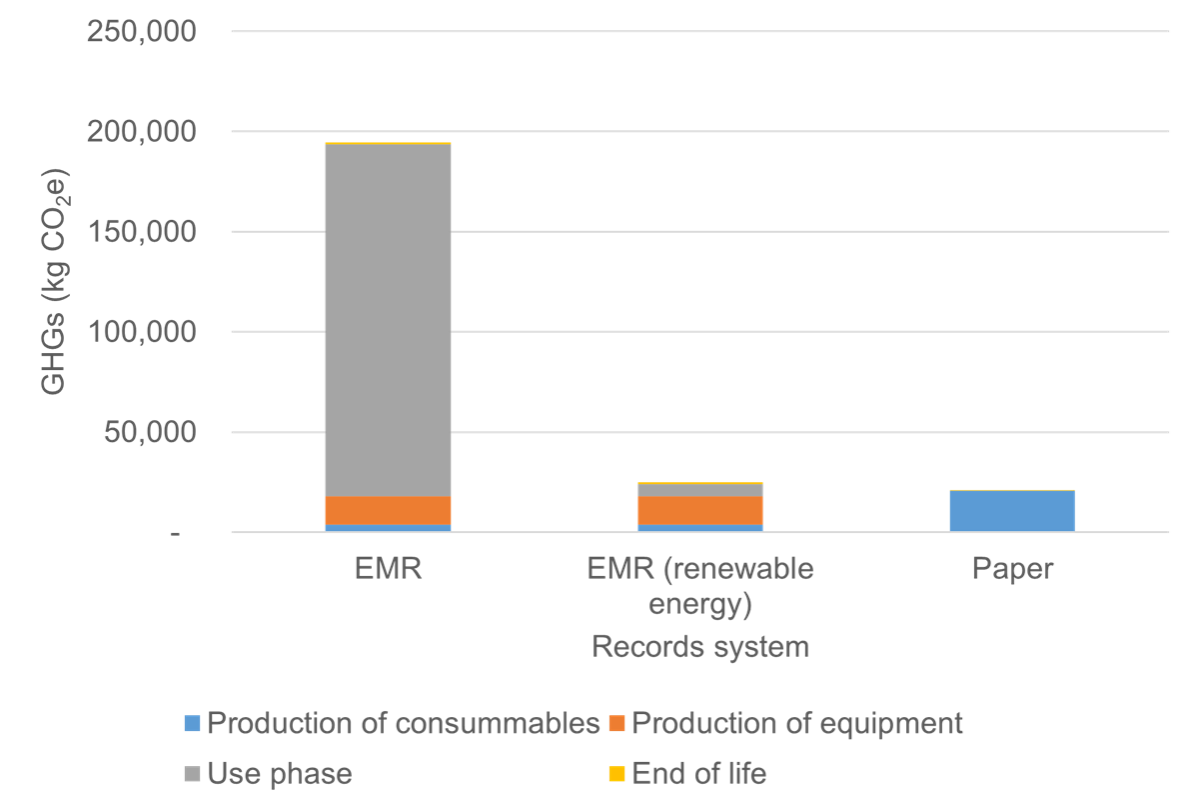
Annual greenhouse gas (GHG) emissions from Aravind’s paper medical record-keeping system and their electronic medical record (EMR) system, with Indian electric grid and with solar power (renewable energy).

The EMR system was found to emit substantially more GHGs than the paper medical record system, shown in Figure 2 and Table S1 in Multimedia Appendix 2. A majority of the EMR system’s emissions (90%, or 175,800 kg CO_2_e per year) are from the electricity used to run the system. The production of computers accounts for 5% of the EMR system’s GHG emissions (10,300 kg CO_2_e per year), and toner production accounts for another 1% (2100 kg CO_2_e per year). Emissions for the paper medical record system are nearly 100% from the production of printed paper (20,700 kg CO_2_e per year). Per patient, both systems generate a small quantity of emissions: 0.361 kg CO_2_e for the EMR system and 0.037kg CO_2_e for the paper system. Results per patient and results for other emissions impact categories can be found in Multimedia Appendix 1.

**Figure 3:**
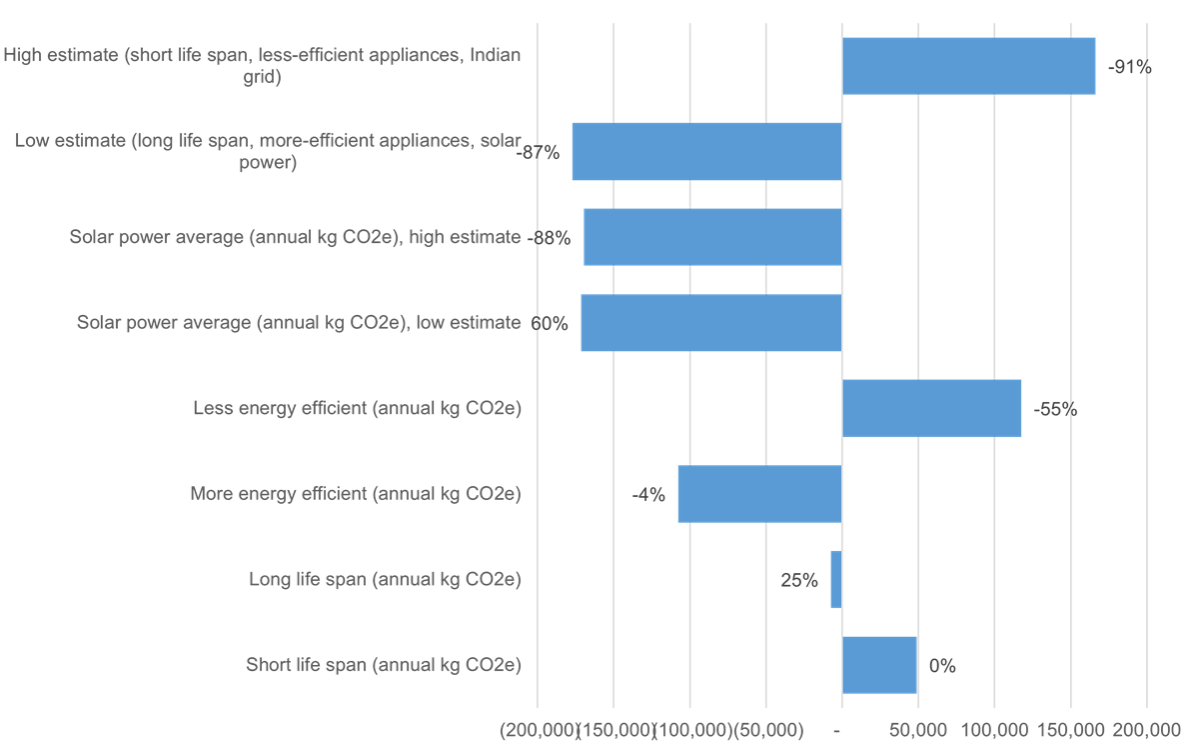
Effects of model and input variability on life cycle greenhouse gas emissions from Aravind’s electronic medical record (EMR) system.

The sensitivity analyses illustrate extreme variability in GHG emissions and the potential for reducing the EMR system’s emissions to a level more comparable to the paper medical record system. Renewable electricity sources seem to be the largest factor, with solar power reducing the EMR system’s emissions by 87%, from 195,000 kg CO_2_e to 24,900 kg CO_2_e (Figure 3). More energy-efficient equipment could reduce emissions by 55% (87,100 kg CO_2_e), while less energy-efficient equipment could increase emissions by 60% (312,000 kg CO_2_e). Aravind is likely already using capital equipment for an optimal life span. Increasing life expectancy (from 10 to 20 years for cables or from 5 to 10 years for computers; Table S3 in Multimedia Appendix 1) could reduce emissions by 4% (187,000 kg CO_2_e), but increasing the frequency of replacement to levels more common in countries like the United States (from 10 to 5 years for cables and from 5 to 1 year for computers) could increase the EMR emissions by 25% (243,000 kg CO_2_e).

**Figure 4:**
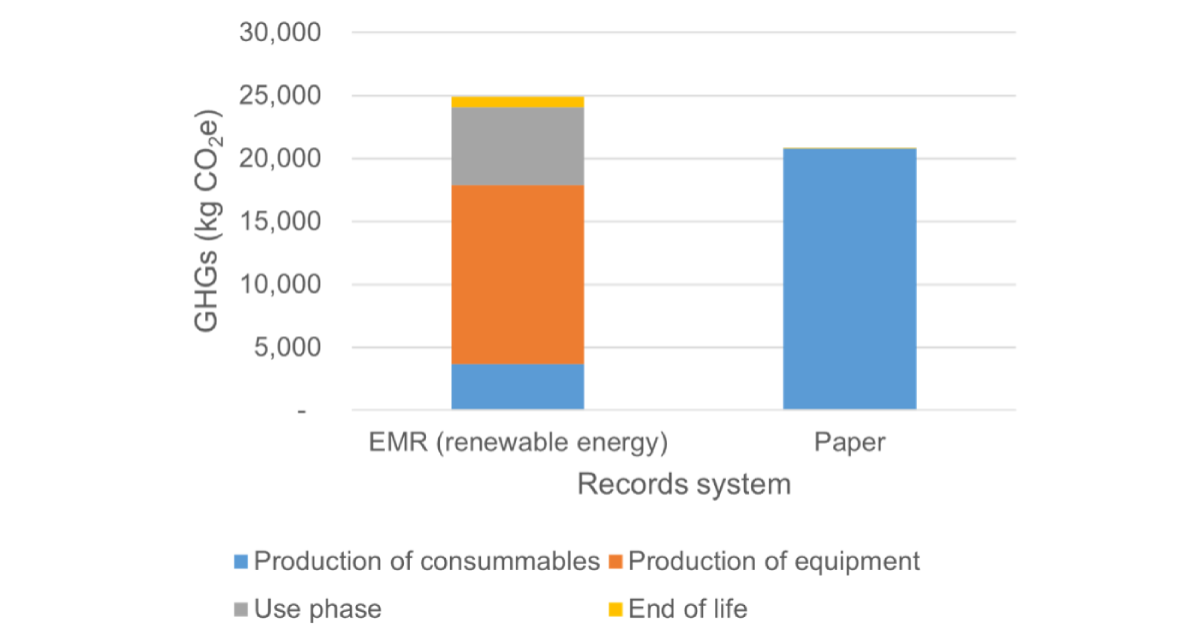
Greater detail of annual Greenhouse Gas (GHG) emissions from Aravind's paper medical record–keeping system and their electronic medical record (EMR) system with renewable energy (solar power).

When assuming the EMR system is powered by solar panels (a renewable energy source), the emissions are more comparable to those of the paper record-keeping system, though the paper record-keeping system still outperforms a renewably powered EMR system (Figure 4). However, the share of emissions shifts when solar power is used, with the electricity required to power the EMR system representing only 25% (6170/24,900 kg CO_2_e) of GHG emissions, rather than 90% 175,796/194,538 kg CO_2_e) (Table S2 in Multimedia Appendix 2). The production of computers becomes the largest source of emissions, at 41%, or 10,300 kg CO_2_e per year (0.02 kg CO_2_e per patient). Production of capital equipment results in 57% (14,220/24,900 kg CO_2_e) of emissions in this scenario, and production of consumables results in 15% (3670/24,900 kg CO_2_e) of GHG emissions.

The MCA assesses model uncertainty and shows that the paper system is preferable to the EMR system on an Indian grid in nearly all environmental impact categories except carcinogenic and noncarcinogenic impacts, where they perform similarly. With solar power, the EMR system’s impacts overlap more with the paper system’s impacts. EMR is comparable to paper record-keeping for carcinogenic and noncarcinogenic emissions categories, where the distribution of EMR impacts is less than paper in about 50% (solar-powered EMR was less than paper record-keeping in 590/1000 runs and 515/1000, respectively) of MCA runs. For air-related categories such as fossil fuel depletion, respiratory effects, global warming, smog formation, and ozone depletion, EMR emissions are lower than paper for about 10% (0/1000, 42/1000, 7/1000, 12/1000, and 362/1000, respectively) of MCA runs (Table S5, Figure S6 in Multimedia Appendix 1). However, for water-related categories such as acidification, eutrophication, and ecotoxicity, the paper record-keeping system outperforms the solar-powered EMR system in nearly 100% (821/1000, 942/1000, and 1000/1000) of MCA runs (95% CI).

## Discussion

### Changing the Footprint of the EMR

Assuming the use of the Indian electric grid, Aravind’s EMR system generates an estimated 195,000 kg CO_2_e per year, or 0.361 kg CO_2_e per patient visit. This far exceeds the GHGs emitted from their paper record-keeping system, with an estimated 20,800 kg CO_2_e per year or 0.037 kg CO_2_e per patient. This is equivalent to the use of 42 passenger vehicles over the course of a year for the EMR system and the use of 4.5 passenger vehicles in the case of the paper record-keeping system.

However, sensitivity analyses show that the effect of electricity sources is a major factor in determining which record-keeping system emits fewer GHGs. Indeed, this study overestimates Aravind’s emissions, as they are using solar panels for about 22% of their electricity needs. Total reliance on renewable energy sources such as solar or wind power will reduce the emissions from the EMR system to a level comparable to paper record-keeping (approximately 24,900 kg CO_2_e). Therefore, beyond the use of solar panels, decarbonizing electric grids could massively reduce impact while also benefiting all other industries reliant on the grid. Of note, proper sourcing of solar photovoltaic systems and other renewable technologies should be a priority, given the labor violations currently in the market [34].

### Study Limitations

This study was conducted 1 year after Aravind implemented their EMR system; however, some residual and unnecessary practices likely remained from the paper-record-keeping system. At the time of this study, Aravind still used a mix of paper and electronic materials. Switching entirely away from paper may result in increased emissions from computer equipment or electricity, but it could reduce the remaining impacts from paper and toner production. A longer-term follow-up study may help assess environmental impacts after the EMR system has been fully implemented [35]. Examples of fuller use of the EMR system include barcode scanners for noting sterilization, devices that allow patients to sign consent forms digitally, or increased training for staff to increase their comfort levels with the EMR system.

This study was conducted at a single institution, and the EMR system set up here may be unique to this setting. We have included sensitivity analyses in part to account for variation in practice, but this study’s results may not be translatable to other settings. In addition, some elements of both systems were not included in this study. The paper record-keeping system did use some computers and printing, which we did not account for. We have also made assumptions around the life spans of EMR technologies, EMR energy use, and the number of consumables used in each system, which can change the emissions profiles of both systems.

### The Unmodeled Environmental Impacts of EMRs

Regardless of electricity source, implementing EMRs, particularly in emerging and expanding health systems in LMICs, has a variety of benefits that are important to consider and have not been captured in this LCA. EMRs improve patient overall care delivery outcomes through enhanced care coordination, enabled data sharing, and decreased clinical errors. Providing the right care to the right patient at the right time ensures emissions from medical practice are not unnecessary [36]. One clinical error that also causes significant waste and unnecessary emissions, ordering duplicative lab tests, has been significantly eliminated in health systems after the adoption of EMRs [37]. Simultaneously, EMRs can increase efficiencies within hospitals and reduce costs in health care systems [7,8]. Aravind was able to reclaim hospital space previously allocated to record storage, facilitating more patient care. In addition, 50% of the staff needed for medical record keeping were reassigned to other critical tasks.

There are other environmental impacts of the transition to EMRs that are not captured by this LCA, a primary one being facilitating telemedicine through enabling the digital transfer of information between patient and doctor. Previous evidence in the US context suggests that transitioning away from paper record-keeping can reduce deforestation from paper production and decrease transportation to hospitals due to increased telemedicine visits or email correspondences [38]. This supports a sizable body of research from high-income countries that points to telemedicine reducing the carbon footprint of appointments, primarily by reducing transport-associated emissions, although the exact carbon footprint savings can vary widely depending on the context [39,40]. Supporting telemedicine has a multitude of other benefits, particularly for LMICs, as the expansion of telemedicine improves access for many vulnerable and geographically remote patients. Since implementing an EMR system, Aravind has created a network of clinics that videoconference with physicians in the main hospital, enabling greater access and triage for patients while maximizing the time and reach of clinicians. Likewise, it has reduced travel and travel-related environmental impacts for providing necessary care.

### Conclusion

EMRs have the potential to transform how health care is delivered in LMICs. However, EMRs have an environmental footprint that should be considered, given the devastating health consequences of climate change. When implementing an EMR system, health care systems should use energy-efficient technologies wherever possible and develop processes and maintenance protocols to increase the life span of these products. Moreover, investing in strategies to decarbonize electric grids is essential to ensuring that health care expansion is conducted more sustainably. This could be done locally by individual hospitals through the installation of solar or wind power or more strategically at a national level, which would have greater influence on other industries as well. Improving the environmental performance of EMRs is an important aspect of making healthcare safe and accessible across the world.

## Appendix

Multimedia Appendix 1 Supplemental information (tables and figures).

Multimedia Appendix 2 Supplementary tables.

## Abbreviations

CO2e: carbon dioxide equivalents
EMR: electronic medical record
GHG: greenhouse gas
ISO: International Organization for Standardization
LCA: life cycle assessment
LCI: life cycle inventory
LCIA: life cycle impact assessment
LMICs: low- and middle-income countryies
MCA: Monte Carlo assessment
TRACI: Tool for Reduction and Assessment of Chemicals and Other Environmental Impacts

## Data Availability

All data generated or analyzed during this study are included in this published article and its supplementary information files.
