# Supplementary material for: The Environmental Impacts of Electronic Medical Records Versus Paper Records at a Large Eye Hospital in India: Life Cycle Assessment Study
Source: J Med Internet Res. 2024 Feb 6;26:e42140. doi: 10.2196/42140 (PMC10879968; doi:10.2196/42140)
Supplement: Multimedia Appendix 1 [file jmir_v26i1e42140_app1.docx]

# Title:

Comparing the Environmental Impacts of Electronic Medical Records and Paper Records at a Large Eye Hospital in India

Contents

Title: 1

Model Inputs 3

Table S1:Model inputs, Unit Process selection, and data sources 3

GHG Emissions from Solar-Powered EMR 6

Table S2: Life Cycle GHG Emissions from EMR powered by solar PV compared to Paper recordkeeping 6

Figure S1: Breakdown of life cycle GHG emissions from EMR powered by solar 7

Figure S2: Breakdown of life cycle GHG emissions from the paper recordkeeping system 8

Figure S3: Comparison of EMR with solar power compared to the paper record-keeping system 9

Sensitivity Analyses 10

Table S3:Assumptions for Sensitivity Analyses of the Electronic Medical Record, including lifespan estimates, equipment power draw and duration of use 10

Figure S4: GHG Impacts of lifespan allocations for EMR capital equipment 11

Figure S5: Impact of Electricity Mix and Equipment Efficiency on use phase GHG emissions 12

Results in other impact categories 12

Table S4: LCA Results for all impact categories* 12

Supplemental Information: Monte Carlo Assessment 14

Table S5: Monte Carlo Results comparing Aravind's Paper System (B) to their EMR (A) assuming Indian electric grid mix 14

Table S6: Monte Carlo Results comparing Aravind's Paper System (B) to their EMR (A) assuming solar power 14

Figure S6: Monte Carlo Results comparing Aravind's 2016 paper recordkeeping system to their EMR in 2019 (assuming Indian electric grid mix); 1000 runs at 95% confidence interval; A = Electronic Medical Record, B = Paper Record-Keeping; A<B = blue color 16

Figure S7: Monte Carlo Results comparing Aravind's 2016 paper recordkeeping system to their EMR in 2019 (assuming all solar power); 1000 runs at 95% confidence interval; A = Electronic Medical Record with solar power, B = Paper Record-Keeping; A<B = blue color 16

References 16

# Model Inputs

## Table S1:Model inputs, Unit Process selection, and data sources

| **Electronic Medical Record System (2019)** | | | | |
| --- | --- | --- | --- | --- |
| *LC Phase* | *Inputs* | *Annual Quantity (#)* | *Unit Processes Used (Ecoinvent v3)* | *Data Collection* |
| Production | Computers | 237 | Computer, desktop, without screen {GLO}\| market for \| Cut-off, U | Primary: purchase records; Secondary: product specifications, existing literature |
|  | Screens | 24 | Display, liquid crystal, 17 inches {GLO}\| market for \| Cut-off, U | Primary: purchase records; Secondary: product specifications, existing literature |
|  | Scanners | 13 | Printer, laser, black/white {GLO}\| market for \| Cut-off, U | Primary: purchase records; Secondary: product specifications |
|  | Printers | 35 | Printer, laser, black/white {GLO}\| market for \| Cut-off, U | Primary: purchase records; Secondary: product specifications |
|  | Routers, Switches | 68 | Router, internet {GLO}\| market for \| Cut-off, U | Primary: purchase records; Secondary: product specifications |
|  | Cables and Chords | 1,170 | Assumed density of 0.45 kg/m and average of: -Cable, connector for computer, without plugs {GLO}\| market for \| Cut-off, U -Cable, data cable in infrastructure {GLO}\| market for \| Cut-off, U -Cable, network cable, category 5, without plugs {GLO}\| market for \| Cut-off, U -Cable, printer cable, without plugs {GLO}\| market for \| Cut-off, U | Primary: purchase records; Secondary: product specifications |
|  | Misc. Equipment (racks and power strips) | 890 | Combination of following, depending on item: Polyethylene, high density, granulate {GLO}\| market for \| Cut-off, U Polyurethane, flexible foam {RER}\| market for polyurethane, flexible foam \| Cut-off, U Steel, unalloyed {GLO}\| market for \| Cut-off, U Beryllium {GLO}\| market for beryllium \| Cut-off, U Gold {GLO}\| market for \| Cut-off, U Nylon 6-6 {RoW}\| market for nylon 6-6 \| Cut-off, U Flat glass, uncoated {RoW}\| market for flat glass, uncoated \| Cut-off, U Copper, cathode {GLO}\| market for \| Cut-off, U | Primary: purchase records; Secondary: product specifications |
|  | Paper | 251,180 | Paper, woodfree, coated {RoW}\| market for \| Cut-off, U | Primary: purchase records; Secondary: product specifications |
|  | Toner | 157 | Toner module, laser printer, black/white {GLO}\| market for \| Cut-off, U | Primary: purchase records; Secondary: product specifications |
| Use | Maintenance/Warranty Service | 6 | NONE | Primary: purchase records |
|  | Electricity | 114,796 | Electricity, low voltage {IN}\| market group for electricity, low voltage \| Cut-off, U Alternate: Electricity, low voltage {IN-TN}\| electricity production, photovoltaic, 3kWp slanted-roof installation, multi-Si, panel, mounted \| Cut-off, U Alternate: Electricity, low voltage {US}\| market group for \| Cut-off, U | Primary: direct measurement with watt meter; Secondary: product specifications, existing literature |
|  | Cloud Storage | 500 | Electricity, low voltage {IN}\| market group for electricity, low voltage \| Cut-off, U Alternate: Electricity, low voltage {IN-TN}\| electricity production, photovoltaic, 3kWp slanted-roof installation, multi-Si, panel, mounted \| Cut-off, U Alternate: Electricity, low voltage {US}\| market group for \| Cut-off, U | Secondary: product specifications, existing literature |
| EOL | E-waste disposal | 1,754 | Combination of following, depending on item: -Used cable {GLO}\| market for \| Cut-off, U -Used desktop computer {GLO}\| market for \| Cut-off, U -Used liquid crystal display module {RoW}\| market for used liquid crystal display module \| Cut-off, U -Used printer, laser {GLO}\| market for \| Cut-off, U -Waste electric and electronic equipment {GLO}\| market for \| Cut-off, U | Secondary: product specifications |
|  | Paper shredding & recycling | 1,256 | none (assumed no benefit to Aravind) | Primary: disposal records |
|  | MSW (printer cartridges) | 40 | Municipal solid waste {RoW}\| treatment of, sanitary landfill \| Cut-off, U | Secondary: estimate, product specifications, existing literature |
| **Paper Medical Record System (2016)** | | | | |
| *LC Phase* | *Inputs* | *Annual Quantity (#)* | *Unit Processes Used (Ecoinvent v3)* | *Data Collection* |
| Production | Paper (offset printed) | 1,567,903 | Printed paper, offset {CH}\| offset printing, per kg printed paper \| Cut-off, U; modified to use Electricity, low voltage {IN-Southern grid}\| market for electricity, low voltage \| Cut-off, U and Heat, district or industrial, natural gas {RoW}\| market for heat, district or industrial, natural gas \| Cut-off, U; assumed only 10% paper loss in production; contains: Paper, woodfree, coated {RoW}\| market for \| Cut-off, U | Primary: purchase records |
|  | Pens | 3,258 | From literature: Robbins (2014), K Prezi: https://prezi.com/rt0ti5r55vvd/carbon-life-cycle-of-a-wood-pencil/ | Primary: purchase records; Secondary: product specifications, existing literature |
|  | Pencils | 777 | From literature: https://www.ethicalstationery.com/its-just-a-pen-right/ | Primary: purchase records; Secondary: product specifications, existing literature |
| EOL | Paper shredding & recycling | 13,476 | none (assumed no benefit to Aravind) | Primary: disposal records |
|  | MSW (pen, pencil) | 17 | Municipal solid waste {RoW}\| treatment of, sanitary landfill \| Cut-off, U | Secondary: estimate |

# GHG Emissions from Solar-Powered EMR

## Table S2: Life Cycle GHG Emissions from EMR powered by solar PV compared to Paper recordkeeping

## Figure S1: Breakdown of life cycle GHG emissions from EMR powered by solar

## Figure S2: Breakdown of life cycle GHG emissions from the paper recordkeeping system

## Figure S3: Comparison of EMR with solar power compared to the paper record-keeping system

# Sensitivity Analyses

## Table S3:Assumptions for Sensitivity Analyses of the Electronic Medical Record, including lifespan estimates, equipment power draw and duration of use

| Items | Count | Sum of weight, kg (or m for cable) | Sum of Costs (INR) | Avg. Lifespan | Short Lifespan | Long Lifespan | Avg. W | Low W | High W | Off W | Hours On (weekday - 6 days per week) | Hours On (weekend - 1 day per week) |
| --- | --- | --- | --- | --- | --- | --- | --- | --- | --- | --- | --- | --- |
| Cables | 1170 | 15,733 | 966,078.27 | 10.0 | 5.0 | 20.0 | - | - | - | - | 10.00 | 10.00 |
| Computer | 237 | 933.66 | 5,405,948.00 | 5.0 | 1.0 | 10.0 | 100.00 | 40.00 | 150.00 | 1.20 | 10.00 | 10.00 |
| IT Support | 6 | 0 | 663,315.00 | 1.0 | - | - | - | - | - | - |  |  |
| Misc | 889.89 | 348.224 | 2,273,839.31 | 10.0 | 5.0 | 20.0 | - | - | - | - | 10.00 | 10.00 |
| Scanner | 13 | 34.25 | 343,695.75 | 4.0 | 1.0 | 10.0 | 4.60 | 1.00 | 6.00 | - | 0.25 | 0.25 |
| Printer | 35 | 208.6 | 476,641.50 | 4.0 | 1.0 | 10.0 | 2.10 | 1.00 | 5.00 | - | 0.25 | 0.25 |
| Routers/  Switches | 68 | 123.52 | 3,832,123.17 | 5.7 | 3.0 | 10.0 | 45.00 | 13.00 | 87.00 | 1.00 | 24.00 | 24.00 |
| Screen | 24 | 106.2 | 434,304.00 | 9.5 | 3.0 | 12.0 | 55.00 | 30.00 | 188.00 | 0.50 | 10.00 | 10.00 |
| Internet (bandwidth) | 500 |  |  |  |  |  |  | - | - | - | 24.00 | 24.00 |
| *Grand Total* | *2442.89* |  | *14395945* |  |  |  |  |  |  |  |  |  |

## Figure S4: GHG Impacts of lifespan allocations for EMR capital equipment

## Figure S5: Impact of Electricity Mix and Equipment Efficiency on use phase GHG emissions

# Results in other impact categories

## Table S4: LCA Results for all impact categories*

*does NOT include pen or pencil manufacturing in the Paper system NOR disposal pathways for either system.

| Impact category | Unit | Aravind EMR | Aravind EMR with Solar Power | Aravind Paper |
| --- | --- | --- | --- | --- |
| Ozone depletion | kg CFC-11 eq | 0.004239 | 0.002398 | 0.001569 |
| Global warming | kg CO2 eq | 193688.1 | 24055.09 | 20725.86 |
| Smog | kg O3 eq | 11169.71 | 1775.002 | 1457.623 |
| Acidification | kg SO2 eq | 888.3529 | 165.4069 | 100.2997 |
| Eutrophication | kg N eq | 870.7529 | 187.6616 | 105.9123 |
| Carcinogenics | CTUh | 0.016875 | 0.00644 | 0.002225 |
| Non carcinogenics | CTUh | 0.076773 | 0.037126 | 0.007512 |
| Respiratory effects | kg PM2.5 eq | 350.3936 | 34.74086 | 27.438 |
| Ecotoxicity | CTUe | 5042952 | 3902303 | 328557.9 |
| Fossil fuel depletion | MJ surplus | 57157.28 | 24256.9 | 20639.25 |

# Supplemental Information: Monte Carlo Assessment

The Monte Carlo Assessments (MCA) were run through SimaPro 9.3.0.2 [31] using inventory data as described in the manuscript. Notably, these results EXCLUDE the production of pens and pencils from the paper recordkeeping system, as these GHG values were pulled from previous literature, and the disposal impacts in both systems. As these particular elements were so small relative to total GHG emissions, they are not expected to significantly impact the results of the MCA. All MCAs contained 1000 runs and are reported with 95% confidence intervals.

## Table S5: Monte Carlo Results comparing Aravind's Paper System (B) to their EMR (A) assuming Indian electric grid mix

| Title: | Uncertainty analysis of 1 p 'Aravind EMR' (A) minus 1 p 'Aravind Paper' (B),  Method: TRACI 2.1 V1.06 / US 2008 , confidence interval: 95 % | | | | | | | |
| --- | --- | --- | --- | --- | --- | --- | --- | --- |
| Indicator: | Characterization | |  |  |  |  |  |  |
| Impact category | A >= B | Mean | Median | SD | CV | 2.5% | 97.5% | SEM |
| Acidification | 100 | 791.4577 | 776.656 | 95.65064 | 12.08538 | 653.3128 | 1035.628 | 3.024739 |
| Carcinogenics | 78.4 | 0.014198 | 0.011753 | 0.027299 | 192.2702 | -0.01493 | 0.055017 | 0.000863 |
| Ecotoxicity | 100 | 4721600 | 4638112 | 950667.1 | 20.13443 | 3097687 | 6844535 | 30062.73 |
| Eutrophication | 100 | 768.4571 | 654.5605 | 452.7676 | 58.91905 | 302.5018 | 1913.904 | 14.31777 |
| Fossil fuel depletion | 100 | 36783.84 | 36668.06 | 3878.646 | 10.54443 | 29276.16 | 44779.22 | 122.6536 |
| Global warming | 100 | 173344.8 | 172462.1 | 16383.54 | 9.451415 | 146236.6 | 209611.1 | 518.0929 |
| Non carcinogenics | 49.6 | 0.017549 | -0.02377 | 1.531697 | 8728.082 | -2.96222 | 3.014281 | 0.048437 |
| Ozone depletion | 100 | 0.002758 | 0.00268 | 0.000528 | 19.14518 | 0.001958 | 0.003947 | 1.67E-05 |
| Respiratory effects | 100 | 323.328 | 322.4283 | 24.47729 | 7.570423 | 277.9393 | 374.2501 | 0.77404 |
| Smog | 100 | 9810.587 | 9738.436 | 872.068 | 8.88905 | 8194.327 | 11760.57 | 27.57721 |

## Table S6: Monte Carlo Results comparing Aravind's Paper System (B) to their EMR (A) assuming solar power

| Title: | Uncertainty analysis of 1 p 'Aravind EMR - solar' (A) minus 1 p 'Aravind Paper' (B),  Method: TRACI 2.1 V1.06 / US 2008 , confidence interval: 95 % | | | | | | | |
| --- | --- | --- | --- | --- | --- | --- | --- | --- |
| Indicator: | Characterization |  |  |  |  |  |  |  |
| Impact category | A >= B | Mean | Median | SD | CV | 2.5% | 97.5% | SEM |
| Acidification | 100 | 67.7236 | 68.95761 | 16.16379 | 23.8673 | 30.4738 | 98.49326 | 0.511144 |
| Carcinogenics | 63.7 | 0.004295 | 0.004061 | 0.013134 | 305.7991 | -0.02082 | 0.031968 | 0.000415 |
| Ecotoxicity | 100 | 3597923 | 3565731 | 783200.8 | 21.76814 | 2218156 | 5357703 | 24766.98 |
| Eutrophication | 99.7 | 82.82219 | 82.37499 | 29.14697 | 35.19222 | 24.65418 | 143.8363 | 0.921708 |
| Fossil fuel depletion | 96.5 | 3665.112 | 3527.943 | 2175.536 | 59.35795 | -307.595 | 8135.75 | 68.79648 |
| Global warming | 89.5 | 3356.648 | 3489.651 | 2814.169 | 83.83864 | -2591.3 | 9026.49 | 88.99182 |
| Non carcinogenics | 49.5 | 0.027396 | -0.01499 | 1.529884 | 5584.431 | -3.00614 | 3.185523 | 0.048379 |
| Ozone depletion | 99.9 | 0.000917 | 0.000902 | 0.000284 | 30.97966 | 0.000422 | 0.001516 | 8.98E-06 |
| Respiratory effects | 92.8 | 7.43104 | 7.634011 | 4.995549 | 67.22544 | -2.71164 | 16.55102 | 0.157973 |
| Smog | 96 | 380.7558 | 387.7503 | 207.296 | 54.44328 | -63.5451 | 772.0451 | 6.555274 |


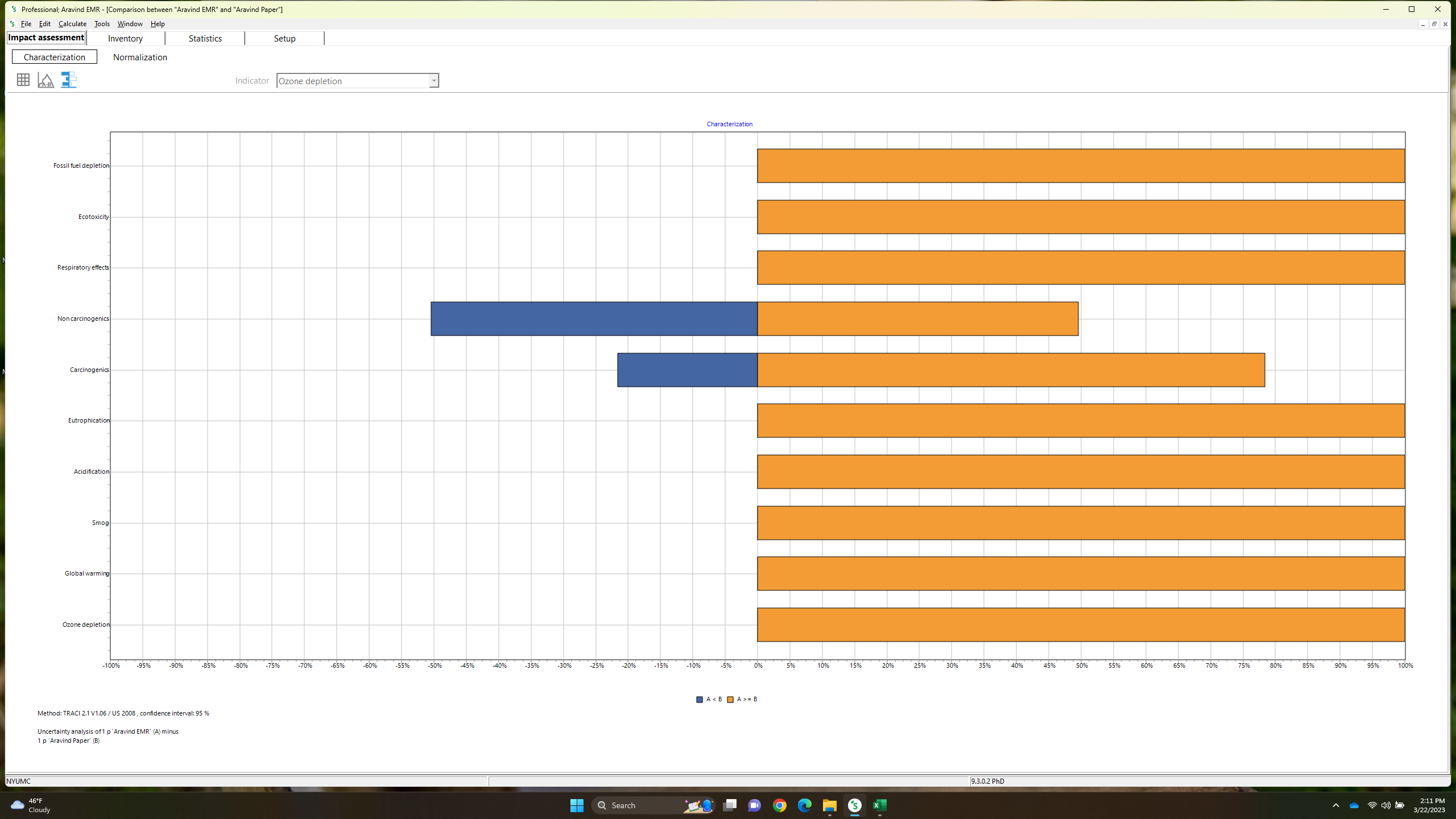


## Figure S6: Monte Carlo Results comparing Aravind's 2016 paper recordkeeping system to their EMR in 2019 (assuming Indian electric grid mix); 1000 runs at 95% confidence interval; A = Electronic Medical Record, B = Paper Record-Keeping; A<B = blue color


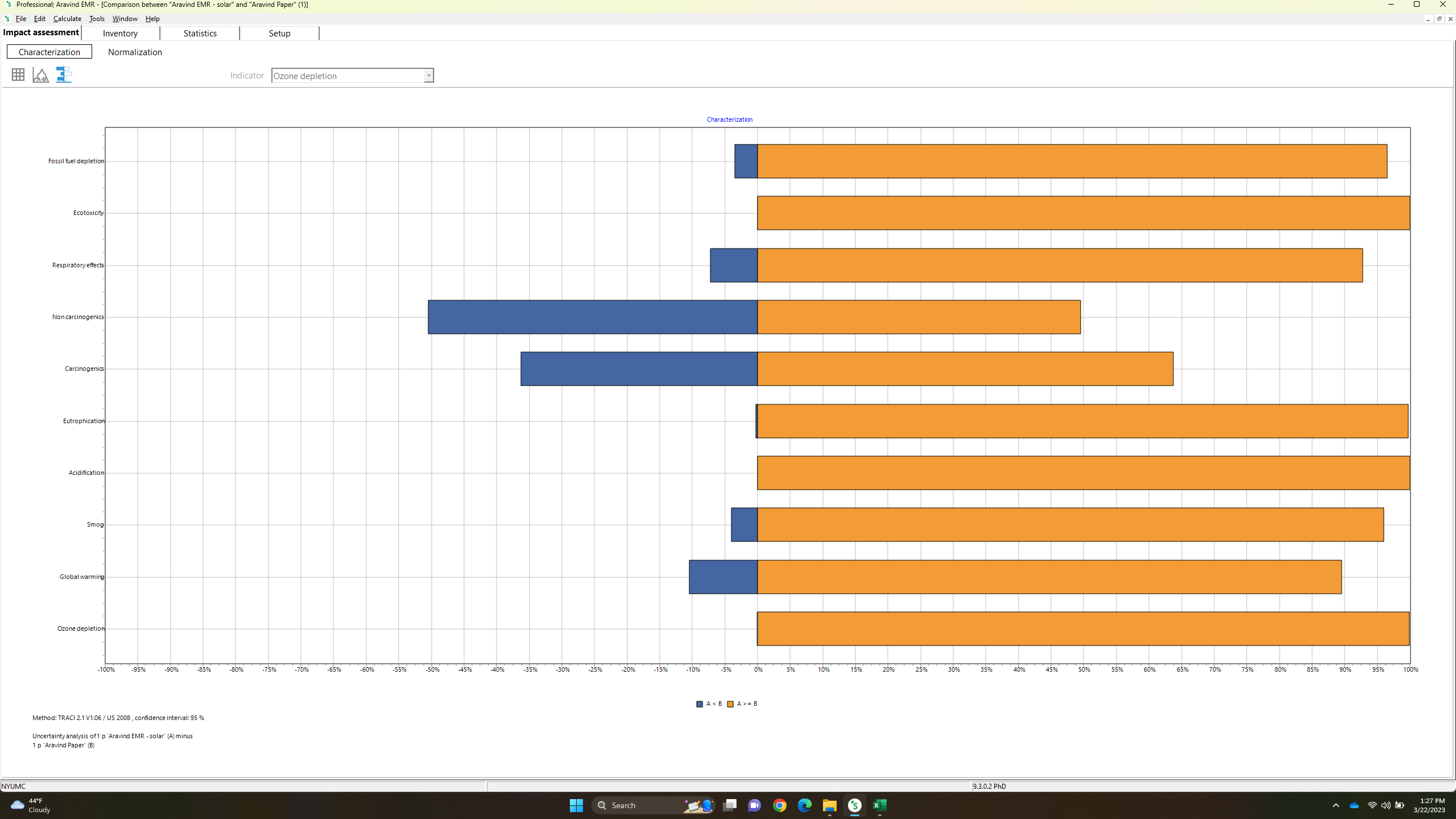


## Figure S7: Monte Carlo Results comparing Aravind's 2016 paper recordkeeping system to their EMR in 2019 (assuming all solar power); 1000 runs at 95% confidence interval; A = Electronic Medical Record with solar power, B = Paper Record-Keeping; A<B = blue color

# References

31. *SimaPro 9.3.0.2* [computer program]. Version 9.3.0.2. Amersfoort, Netherlands: PRé Consultants; 2022.
