# Supplementary material for: The Environmental Impacts of Electronic Medical Records Versus Paper Records at a Large Eye Hospital in India: Life Cycle Assessment Study
Source: J Med Internet Res. 2024 Feb 6;26:e42140. doi: 10.2196/42140 (PMC10879968; doi:10.2196/42140)
Supplement: Multimedia Appendix 2 [file jmir_v26i1e42140_app2.docx]

**Table S1.** Life cycle greenhouse gas emissions (GHGs) from Aravind Eye Care System's electronic medical record (EMR) system and their paper medical record-keeping, model inputs and results in kg CO2e; LC = life cycle, EOL = end of life; INR/Rs – Indian rupee (monetary value); MSW = municipal solid waste; * scanners modeled as printers in the LCA; † toner quantity estimated from printer types rather than toner purchases; ‡ quantity estimated from purchased paper from an offset printer, unit process modified to Indian electric grid.

| **Electronic Medical Record System (2019)** | | | | | | | | | | |
| --- | --- | --- | --- | --- | --- | --- | --- | --- | --- | --- |
| *LC Phase* | *Inputs* | *Quantity (annual)* | *Unit (units, kWh, or kg)* | *Quantity per patient* | *UpFront Purchase Price (Rs or INR)* | *Annual Purchase Price (Rs or INR)* | *Price (INR) per patient* | *GHGs Annually (kg CO2e)* | *GHGs per patient (kg CO2e)* | *%* |
| Production | Computers | 237 | items | 0.00 | 5,405,948 | 1,081,190 | 2.01 | 10,300 | 0.019 | 5.3% |
|  | Screens | 24 | items | 0.00 | 434,304 | 45,716 | 0.08 | 892 | 0.002 | 0.5% |
|  | Scanners* | 13 | items | 0.00 | 343,696 | 85,924 | 0.16 | 198 | 0.000 | 0.1% |
|  | Printers | 35 | items | 0.00 | 476,642 | 83,621 | 0.16 | 534 | 0.001 | 0.3% |
|  | Routers, Switches | 68 | items | 0.00 | 3,832,123 | 672,302 | 1.25 | 1,250 | 0.002 | 0.6% |
|  | Cables and Chords | 1,170 | items | 0.00 | 966,078 | 96,608 | 0.18 | 712 | 0.001 | 0.4% |
|  | Misc. Equipment (racks and power strips) | 890 | items | 0.00 | 2,273,839 | 227,384 | 0.42 | 314 | 0.001 | 0.2% |
|  | Paper | 251,180 | sheets | 0.47 | 204,887 | 204,887 | 0.38 | 1,540 | 0.003 | 0.8% |
|  | Toner† | 157 | cartridges | 0.00 | 337,326 | 337,326 | 0.63 | 2,120 | 0.004 | 1.1% |
| Use | Maintenance/Warranty Service | 6 | contracts | 0.00 | 663,315 | 227,384 | 0.42 | - | - | 0% |
|  | Electricity | 114,796 | kWh | 0.21 |  | - | - | 176,000 | 0.327 | 90% |
|  | Cloud Storage | 500 | GB | 0.00 |  | - | - | 5 |  | 0% |
| EOL | E-waste disposal | 1754 | kg | 0.00 |  | - | - | 830 | 0.002 | 0% |
|  | Paper shredding & recycling | 1,256 | kg | 0.00 |  | - | - | - | - | 0% |
|  | MSW (printer cartridges) | 40 | kg | 0.00 |  | - | - | 20 | 0.000 | 0% |
| ***TOTAL*** | | | | | ***14,938,158*** | ***3,062,342*** | ***5.7*** | ***195,000*** | ***0.361*** | ***100%*** |
| subtotal | ***production of equipment*** | | | | | | | ***14,200*** | ***0.026*** | ***7%*** |
| subtotal | ***production of consumable***  ***es*** | | | | | | | ***3,670*** | ***0.007*** | ***2%*** |
| subtotal | ***use phase*** | | | | | | | ***176,000*** | ***0.327*** | ***90%*** |
| subtotal | ***EOL/Disposal*** | | | | | | | ***850*** | ***0.002*** | ***0%*** |
| **Paper Medical Record System (2016)** | | | | | | | | | | |
| *LC Phase* | *Inputs* | *Quantity (annual)* | *Unit (#, kWh, or kg)* | *Quantity per patient* | *UpFront Purchase Price (Rs or INR)* | *Annual Purchase Price (Rs or INR)* | *Price per patient* | *GHGs Annually (kg CO2e)* | *GHGs per patient (kg CO2e)* | *%* |
| Production | Paper (offset printed)‡ | 1,567,903 | sheets | 2.76 | 2,159,052 | 2,159,052 | 3.79 | 20,700 | 0.036 | 100% |
|  | Pens | 3,258 | # | 0.01 | 7,245 | 7,245 | 0.01 | 70 | 0.000 | 0% |
|  | Pencils | 777 | # | 0.00 | 3,413 | 3,413 | 0.01 | 1 | 0.000 | 0% |
| EOL | Paper shredding & recycling | 13,476 | kg | 0.02 |  | - | - | - | - | 0% |
|  | MSW (pen, pencil) | 17 | kg | 0.00 |  | - | - | 9 | 0.000 | 0% |
| ***TOTAL*** | | | | | ***2,169,710*** | ***2,169,710*** | ***3.8*** | ***20,800*** | ***0.037*** | ***100%*** |
| subtotal | ***production of consumables*** | | | | | | | ***20,800*** | ***0.037*** | ***100%*** |
| subtotal | ***EOL/Disposal*** | | | | | | | ***9*** | ***0.000*** | ***0%*** |

**Table S2.** Annual Greenhouse Gas (GHG) emissions from Aravind's electronic medical record (EMR) system modeled with solar power (renewable energy) rather than Indian electric grid, model inputs and results in kg CO2e; LC = life cycle, EOL = end of life; INR/Rs – Indian rupee (monetary value); MSW = municipal solid waste; * scanners modeled as printers in the LCA; † toner quantity estimated from printer types rather than toner purchases.

| **Electronic Medical Record System (2019) WITH RENEWABLE ENERGY** | | | | | | | | | | |
| --- | --- | --- | --- | --- | --- | --- | --- | --- | --- | --- |
| *LC Phase* | *Inputs* | *Quantity (annual)* | *Unit (units, kWh, or kg)* | *Quantity per patient* | *UpFront Purchase Price (Rs or INR)* | *Annual Purchase Price (Rs or INR)* | *Price (INR) per patient* | *GHGs Annually (kg CO2e)* | *GHGs per patient (kg CO2e)* | *%* |
| Production | Computers | 237 | items | 0.00 | 5,405,948 | 1,081,190 | 2.01 | 10,300 | 0.019 | 41.4% |
|  | Screens | 24 | items | 0.00 | 434,304 | 45,716 | 0.08 | 892 | 0.002 | 3.6% |
|  | Scanners* | 13 | items | 0.00 | 343,696 | 85,924 | 0.16 | 198 | 0.000 | 0.8% |
|  | Printers | 35 | items | 0.00 | 476,642 | 83,621 | 0.16 | 534 | 0.001 | 2.1% |
|  | Routers, Switches | 68 | items | 0.00 | 3,832,123 | 672,302 | 1.25 | 1,250 | 0.002 | 5.0% |
|  | Cables and Chords | 1,170 | items | 0.00 | 966,078 | 96,608 | 0.18 | 712 | 0.001 | 2.9% |
|  | Misc. Equipment (racks and power strips) | 890 | items | 0.00 | 2,273,839 | 227,384 | 0.42 | 314 | 0.001 | 1.3% |
|  | Paper | 251,180 | sheets | 0.47 | 204,887 | 204,887 | 0.38 | 1,540 | 0.003 | 6.2% |
|  | Toner† | 157 | cartridges | 0.00 | 337,326 | 337,326 | 0.63 | 2,120 | 0.004 | 8.5% |
| Use | Maintenance/Warranty Service | 6 | contracts | 0.00 | 663,315 | 227,384 | 0.42 | - | - | 0% |
|  | Electricity | 114,796 | kWh | 0.21 |  | - | - | 6,170 | 0.011 | 25% |
|  | Cloud Storage | 500 | GB | 0.00 |  | - | - | 0 |  | 0% |
| EOL | E-waste disposal | 1,754 | kg | 0.00 |  | - | - | 830 | 0.002 | 3% |
|  | Paper shredding & recycling | - | kg | - |  | - | - | - | - | 0% |
|  | MSW (printer cartridges) | 40 | kg | 0.00 |  | - | - | 20 | 0.000 | 0% |
| ***TOTAL*** | | | | | ***14,938,158*** | ***3,062,342*** | ***5.7*** | ***24,900*** | ***0.046*** | ***100%*** |
| Subtotal | ***production of equipment*** | | | | | | | ***14,200*** | ***0.026*** | ***57%*** |
| Subtotal | ***production of consumables*** | | | | | | | ***3,670*** | ***0.007*** | ***15%*** |
| Subtotal | ***use phase*** | | | | | | | ***6,170*** | ***0.011*** | ***25%*** |
| Subtotal | ***EOL/Disposal*** | | | | | | | ***850*** | ***0.002*** | ***3%*** |
| **Paper Medical Record System (2016)** | | | | | | | | | | |
| *LC Phase* | *Inputs* | *Quantity (annual)* | *Unit (#, kWh, or kg)* | *Quantity per patient* | *UpFront Purchase Price (Rs or INR)* | *Annual Purchase Price (Rs or INR)* | *Price per patient* | *GHGs Annually (kg CO2e)* | *GHGs per patient (kg CO2e)* | *%* |
| Production | Paper (offset printed)‡ | 1,567,903 | sheets | 2.76 | 2,159,052 | 2,159,052 | 3.79 | 20,700 | 0.036 | 100% |
|  | Pens | 3,258 | # | 0.01 | 7,245 | 7,245 | 0.01 | 70 | 0.000 | 0% |
|  | Pencils | 777 | # | 0.00 | 3,413 | 3,413 | 0.01 | 1 | 0.000 | 0% |
| EOL | Paper shredding & recycling | 13,476 | kg | 0.02 |  | - | - | - | - | 0% |
|  | MSW (pen, pencil) | 17 | kg | 0.00 |  | - | - | 9 | 0.000 | 0% |
| ***TOTAL*** | | | | | ***2,169,710*** | ***2,169,710*** | ***3.8*** | ***20,800*** | ***0.037*** | ***100%*** |
| Subtotal | ***production of consumables*** | | | | | | | ***20,800*** | ***0.037*** | ***100%*** |
| Subtotal | ***EOL/Disposal*** | | | | | | | ***9*** | ***0.000*** | ***0%*** |
